# An aggressive central giant cell granuloma in a pediatric patient: case report and review of literature

**DOI:** 10.1186/s40463-019-0356-5

**Published:** 2019-07-18

**Authors:** Yiqiao Wang, Andre Le, Dina El Demellawy, Mary Shago, Michael Odell, Stephanie Johnson-Obaseki

**Affiliations:** 1 Faculty of Medicine, University of Ottawa, Ottawa, Canada; 2 Department of Otolaryngology – Head and Neck Surgery, Faculty of Medicine, University of Ottawa, Ottawa, Canada; 3 Department of Pathology, Children’s Hospital of Eastern Ontario, Faculty of Medicine, University of Ottawa, Ottawa, Canada; 4 Department of Paediatric Laboratory Medicine, The Hospital for Sick Children, Toronto, Canada; 5 Department of Laboratory Medicine and Pathobiology, University of Toronto, Toronto, Canada

**Keywords:** Central giant cell granuloma, Mandible, Aggressive, Pediatrics

## Abstract

**Background:**

Central giant cell granulomas are benign tumours of the mandible, presenting in children and young adults. Divided into non- and aggressive subtypes, the aggressive subtype is relatively rare and can occasionally progress rapidly, resulting in significant morbidity.

**Case presentation:**

We present a case of an aggressive central giant cell granuloma (CGCG) in a six year-old female. The lesion originated in the right mandibular ramus and progressed rapidly to involve the condyle. Diagnosis was made using a combination of imaging and pathology. A timely en bloc resection of the hemi-mandible was performed with placement of a reconstructive titanium plate and condylar prosthesis.

**Conclusion:**

Our case demonstrates the importance of considering CGCG in the differential diagnosis of rapidly progressive mandibular lesions in the pediatric population. Prompt diagnosis and management can greatly improve long-term outcomes.

## Background

Central giant cell granuloma (CGCG) is described by the World Health Organization as an intraosseous lesion consisting of cellular fibrous tissue that contains multiple foci of hemorrhage, aggregations of multinucleated giant cells, and some trabeculae of woven bone [1]. These tumours account for 7% of all benign tumours of the mandible, and present with a higher frequency in the mandible than maxilla [2, 3]. There is a slight female predilection, with a peak age incidence range between 10 to 25 years [4, 5]. Aetiology is unknown, however may be related to trauma, inflammatory foci or genetic predisposition [5, 6].

CGCG can be divided into two subtypes, aggressive and non-aggressive [7]. The non-aggressive variant is most common, presenting as a slow-growing, painless lesion with expansion of cortical bone. In contrast, aggressive giant cell granulomas tend to present in younger patients with the following possible features: greater than 5 cm in size, rapid growth, root resorption, tooth displacement leading to malocclusion, cortical bone thinning or perforation, and recurrence after curettage [7–9].

We report an uncommon case of an aggressive CGCG in a 6-year-old female, originating in the right mandibular ramus with extension into the condyle.

## Case presentation

A six year-old, previously healthy female initially presented to the dentist with a cavity involving a right mandibular tooth, which was filled. Two days after the appointment, she developed mild facial swelling on the ipsilateral side. Symptoms progressed over the next week, and antibiotics were prescribed for a presumed odontogenic infection. The lesion was refractory to antibiotics, which resulted in her presentation to the Children’s Hospital of Eastern Ontario (CHEO) Emergency Department.

**Fig. 1:**
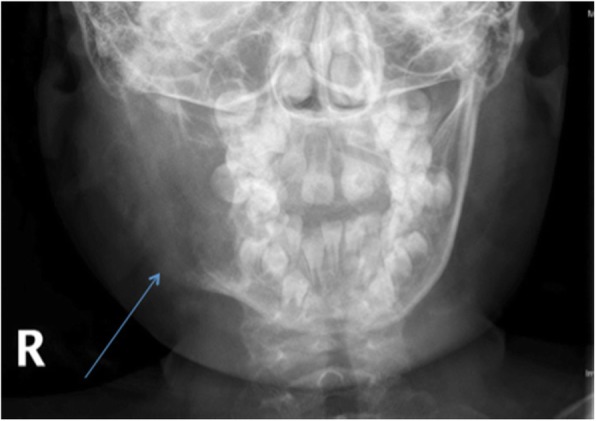
Frontal radiograph of the facial bones at initial presentation to the emergency department. Arrow is pointing to site of lesion

An ultrasound of the face and radiograph of the facial bones was completed (Fig. 1). The radiograph demonstrated a mixed fluid and solid lesion within the right para-mandibular region, measuring 5.8 × 3.4 × 2.9 cm, and with similar consistency to bone (Fig. 1). Similarly, ultrasound demonstrated a large, expansile, lucent, osseous lesion centered in the right mandibular ramus, and described with benign features. The differential diagnoses at the time included giant cell granuloma, dentigerous cyst, radicular cyst, odontogenic keratocyst, and ameloblastoma. In addition, infection could not be completely excluded.

**Fig. 2:**
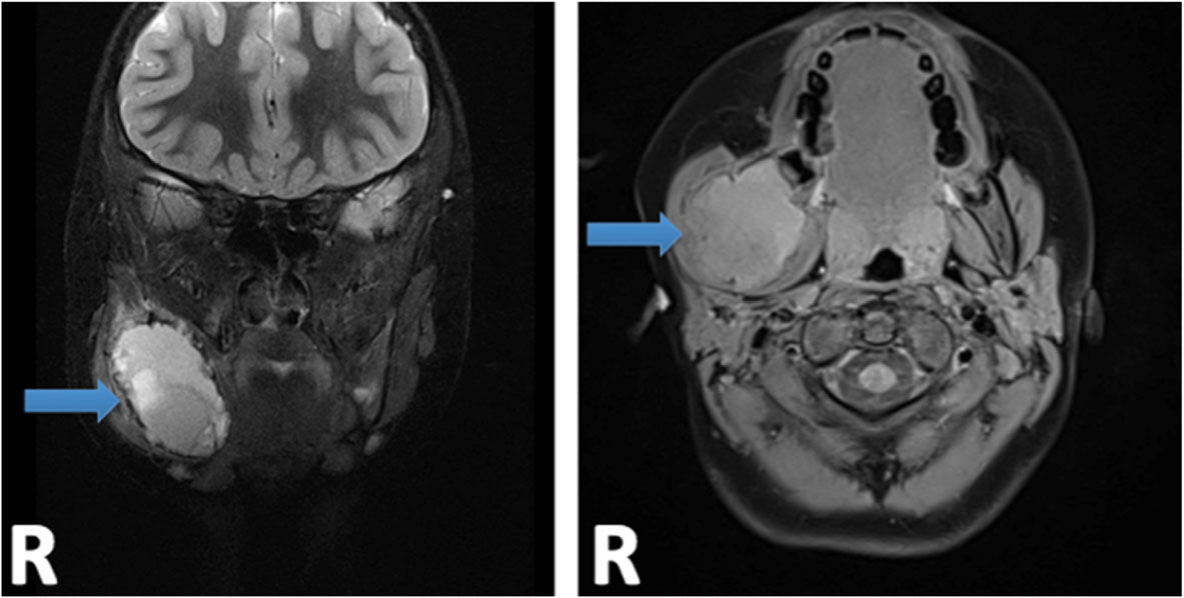
MRI Head following initial presentation to the emergency department with coronal and axial views. Arrows are pointing to site of lesion

**Fig. 3:**
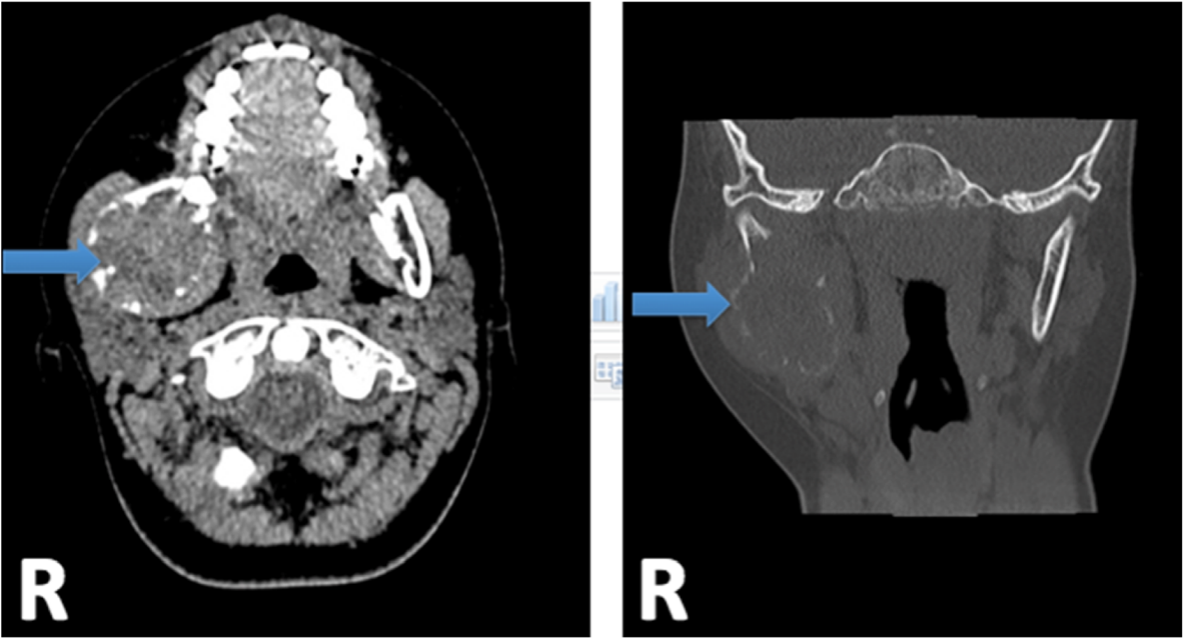
CT Head following initial presentation to the emergency department, with axial and coronal views. Arrows are pointing to site of lesion

The child was discharged home, with an urgent magnetic resonance imaging (MRI) and computed tomography (CT) ordered and performed one day after the emergency visit. MRI of the head demonstrated a cystic lesion centered along the ramus of the right mandible, measuring 3.6 × 3.8 × 4.8 cm (Fig. 2). CT of the head demonstrated a lesion involving the right mandibular ramus, angle and posterior body with involvement of the 2nd mandibular molar (Fig. 3). There was no airway or vascular compromise noted on either imaging modality.

A referral was made to Otolaryngology- Head and Neck Surgery. The patient was taken urgently to the operating room (OR) and a trans-oral biopsy of the mandibular mass was performed. Pathology revealed a multi-cytic lesions. The cysts were devoid of endothelial and epithelial lining and contain areas of hemorrhage. The septa lining the cysts were thick and formed of bland spindle myofibroblasts. Focal hemosiderin deposition and small, unevenly distributed clusters of giant cells were noted. A giant cell granuloma was the favoured diagnosis.

After diagnosis, oral maxillofacial surgery (OMFS) was consulted. The patient was taken again to the OR for enucleation of the tumour, decompression of the right inferior alveolar nerve, and biopsy. The child was to be followed-up by oncology after pathology results returned for consideration of interferon therapy.

**Fig. 4:**
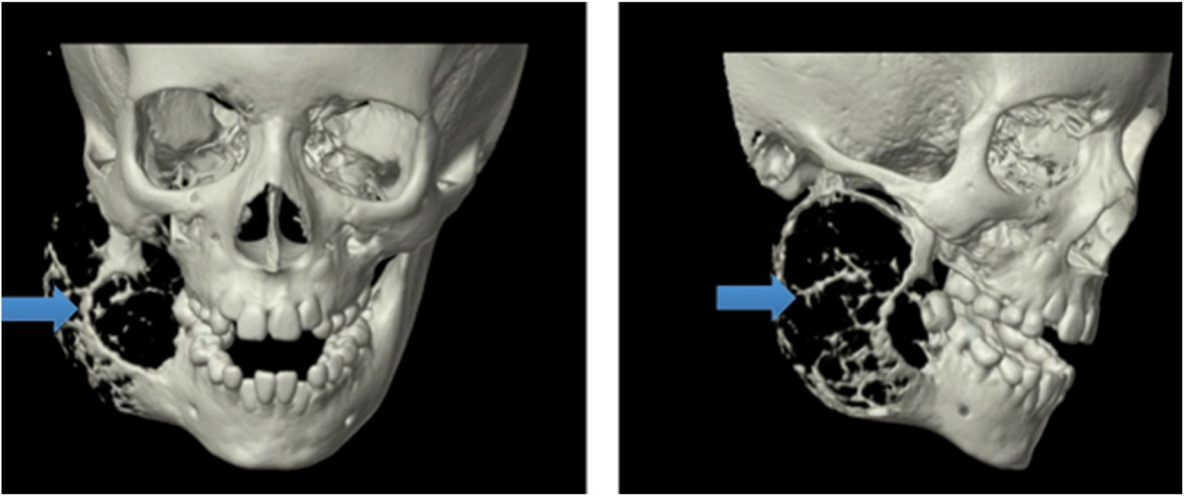
3D CT reconstruction of skull at second presentation to the emergency department. Arrows are pointing to lesion. Significant obliteration of the right hemimandbile including condyle

Sixteen days later, while awaiting follow-up, the patient re-presented to the CHEO ED with facial swelling, pain and an inability to tolerate feeds. Additionally, she complained of numbness of the right tongue, which she had not experienced previously. Repeat CT demonstrated significant growth of the mass to 6.4 × 4.9 × 4.4 cm (Fig. 4).

**Fig. 5:**
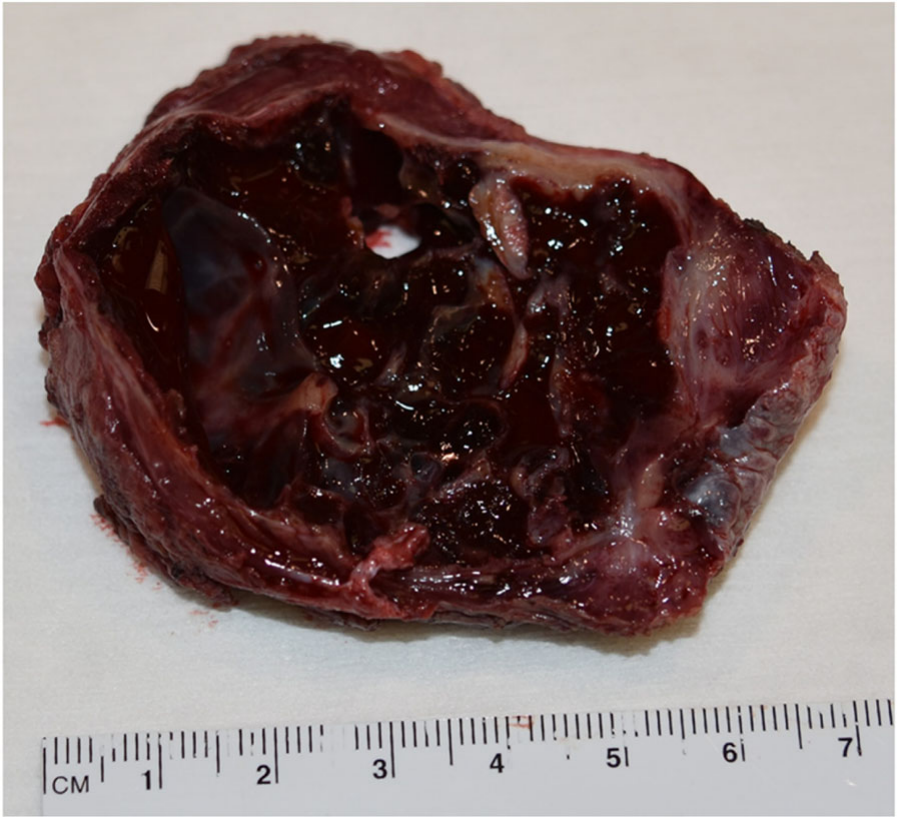
Gross picture of the second excision showing multicystic lesion filled with clotted blood

**Fig. 6:**
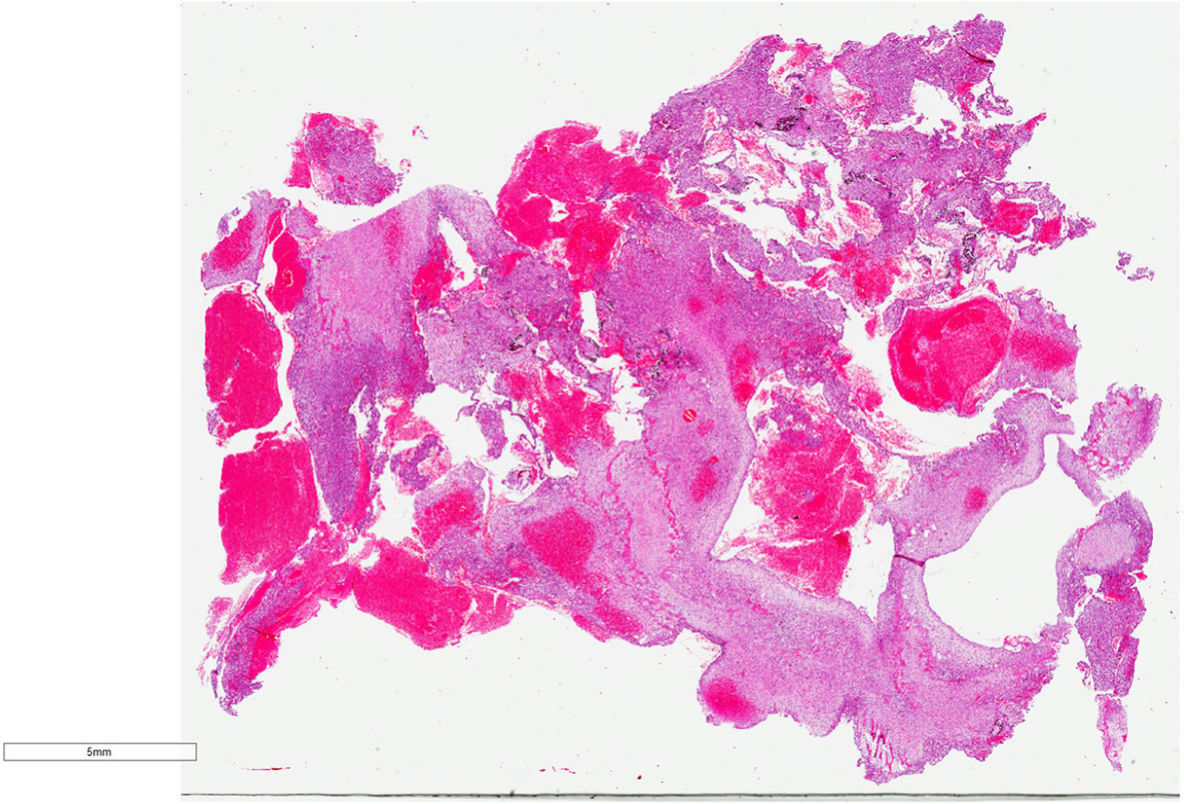
Low power light microscopic view of the lesion showing multicystic spaces filled with blood (H&E X100)

**Fig. 7:**
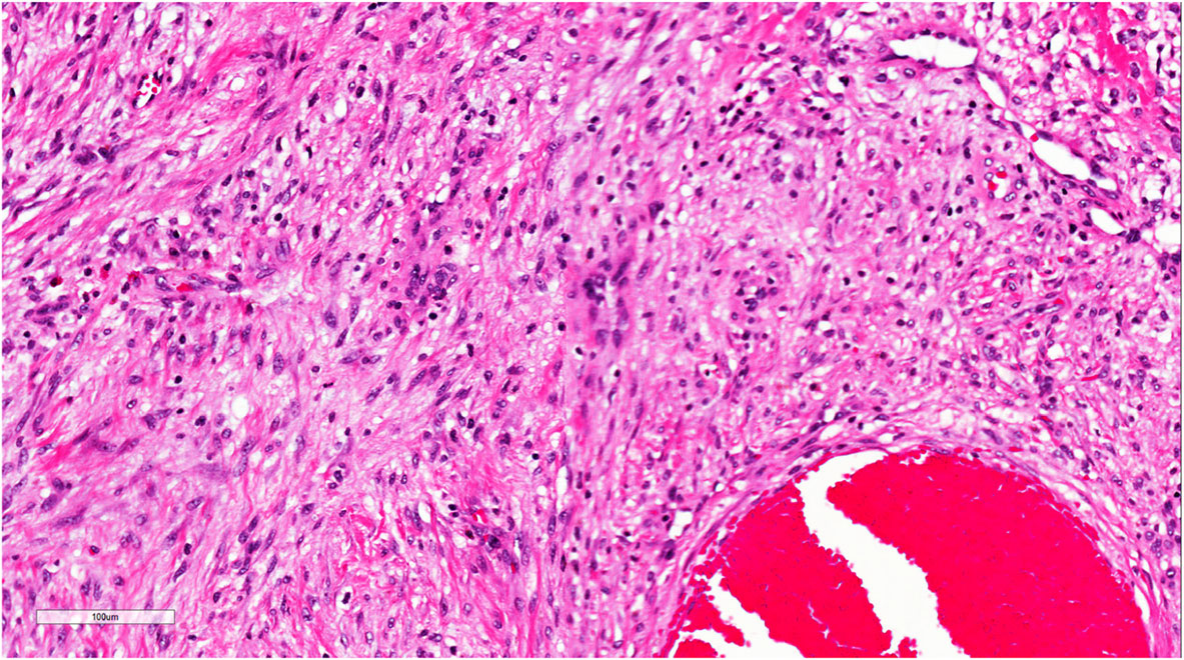
Medium power light microscopic view of the lesion showing fibrous thick septa with reactive woven bony trabeculae. The later are highlighted by back arrow heads (H&E X400)

**Fig. 8:**
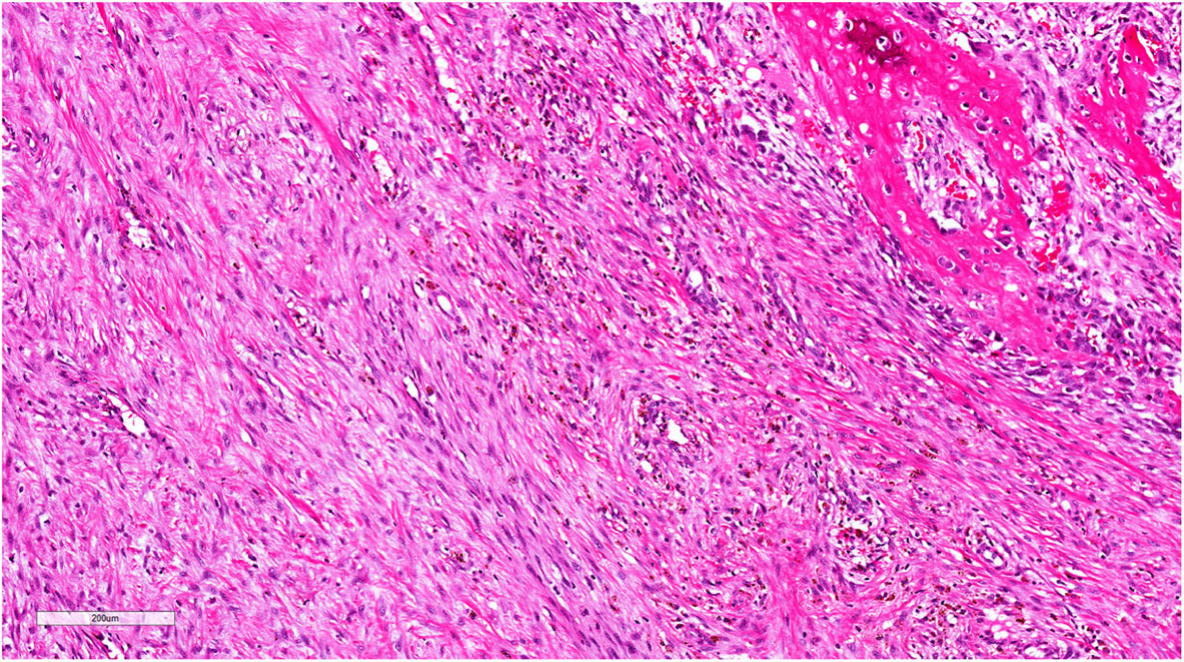
High power light microscopic view of the lesion showing a cystic space and a thick septum formed of myofibroblastic cells. Note the cystic space is devoid of lining (H&E X400)

**Fig. 9:**
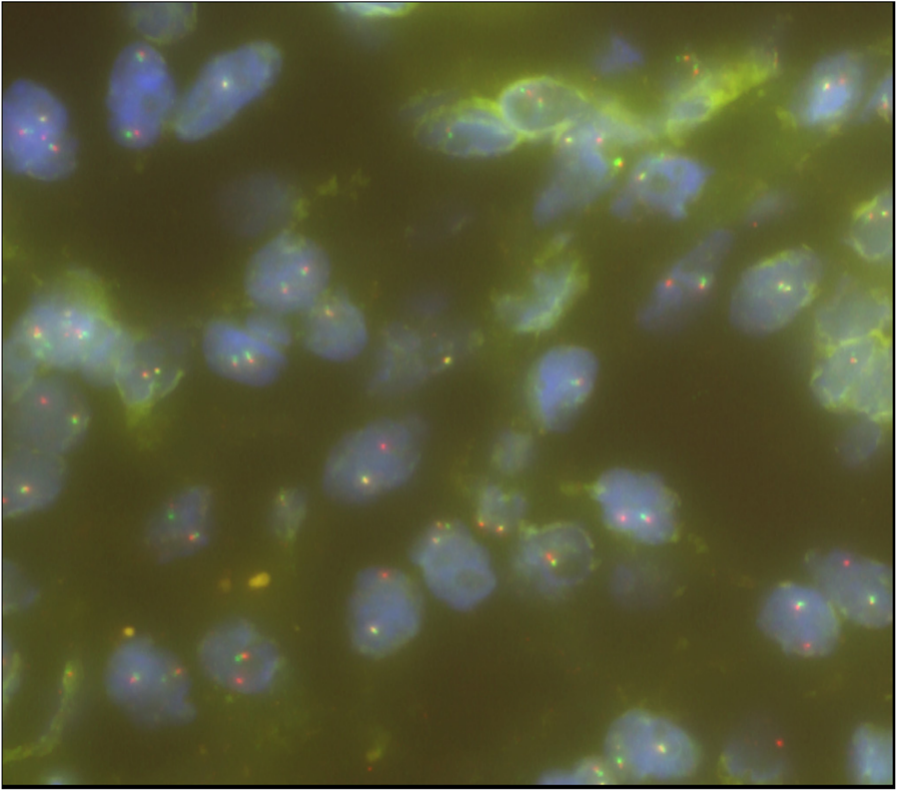
Interphase FISH analysis using a dual colour breakapart probe for the USP6 gene locus (17p13.2; ZytoVision, Bremerhaven, Germany) showing USP6 gene rearrangement in lesional cell nuclei. The abnormal signal pattern has loss of the 5′ USP6 signal with retention of the 3′ USP6 signal

Pathology from the second excisional biopsy showed a lesion with multicystic component filled with clotted blood (Figs. 5 and 6). The cysts were devoid of endothelial and epithelial lining and contained areas of hemorrhage. The septa lining the cysts were thick and formed of bland spindle myofibroblasts (Figs. 7 and 8) and enclosed hemosiderin denoting organizing hemorrhage. Scattered small giant cells with a zonal pattern of distribution were noted. Mitotic activity was sparse with 1 mitotic figure/20 high power fields, identified in the spindle cells. Reactive bone formation was noted at the periphery of the lesion (Fig. 8). The findings supported the original diagnosis of a giant cell granuloma. Interphase FISH analysis performed on a paraffin-embedded tissue slide using a dual colour breakapart probe for the USP6 gene locus (17p13.2) was consistent with USP6 gene rearrangement in 93/200 (46.5%) nuclei (Fig. 9).

The patient was seen by Oncology and the consensus was that interferon therapy would not be warranted unless further resection was performed. Due to the significant size and location of the lesion, the adult Head and Neck Oncology subspecialty service was asked to evaluate the patient. Given the size, patient symptoms, and rapidly growing nature of tumor, the plan was for urgent surgical intervention including a tracheostomy, complete resection of right hemi-mandible and involved buccal mucosa and a complete reconstruction of mandible using a reconstructive titanium plate and condylar prosthesis. The decision to not reconstruct with a bony free tissue flap was made in conjunction with the OMFS team and the adult reconstructive team with the goal to delay the definitive reconstruction and to reassess once the patient was closer to fully grown.

The patient was taken to the OR and surgery proceeded as planned. There were no intraoperative complications. Due to ongoing diagnostic uncertainty, coupled with the malignant clinical behavior of the lesion, a 1.5 cm margin was excised. Postoperatively, the patient was monitored in an intensive care setting. A post-operative day 7 an MRI was obtained, and daily Interferon was started as per oncology at 3,000,000 units subcutaneous daily. The patient was decannulated post-operative day 10 and discharged home after a 13-day admission. Unfortunately she was then admitted on post-operative day 23 with an orocutaneous fistula confirmed via blue dye test. The Interferon at that point was stopped, as there is a known risk of wound dehiscence. She was made NPO and intravenous Cefazolin and Metronidazole were started. She was taken to the operating room for wound debridement and irrigation as well as to facilitate packing of the wound with ribbon gauze. Prior to discharge, packing was changed twice weekly. She was admitted for a total of 32 days and was discharged home on IV Clindamycin as per the Infectious Disease service and feeds via nasogastric tube. She returned to the operating room twice weekly for further packing changes. After 3 further packing changes, the fistula had completely healed and was confirmed to be closed via blue dye test. IV Clindamycin was continued for a total of 6 weeks, and oral Clindamycin then for a further 3 weeks. Further follow-up will include reassessment by the OMFS service and likely exchange to a longer titanium plate, followed by possible definitive reconstruction when she is closer to fully grown.

## Discussion

Our case report describes a rare case of an aggressive CGCG originating in the right mandibular ramus with extension into the condyle in a six year-old child. To our knowledge, only eight other cases of CGCG affecting the mandibular condyle have been presented in literature, and of these, only one occurred in a child [10–17]. Furthermore, only one case required en bloc resection and fibula graft for management [14].

Care should be used to differentiate aggressive and non-aggressive granulomas. In both cases, immunophenotypic and ultrasound findings are similar [18]. Although controversial, in aggressive lesions a higher level of a proliferation marker, Ki-67 may be found [8, 18]. In addition, histomorphometric analysis may show an increased number of giant cells, larger surface area, and greater mitotic activity in the aggressive variant [8]. In our case Ki67 showed a low proliferation index (< 2%) and mitotic figures were rare. Other benign lesions included in the differential diagnosis include: Brown tumors, which are usually multiple lesions and result from increased parathyroid hormone levels, as well as cherubism, which is a congenital disorder causing marked expansion of all 4 quadrants of the jaw. Both of these conditions have similar histopathological features to CGCG [19]. In our case parathyroid hormone levels were not elevated and on imaging there were no other bony or soft tissue lesions identified.

From the pathology prospective our case is interesting as it showed significant cystic areas. CGCG is considered the solid variant of aneurysmal bone cyst, however, aneurysmal bone cystic changes are a finding that can be seen in CGCG. CGCG are usually solid but in a small subset of cases, can show cystic areas with areas identical to aneurysmal bone cyst, as identified in our case. The distinction between CGCG and aneurysmal bone cyst is not feasible particularly on these cases with a dominant and prominent blood-filled cysts. Our case supports the speculation that CGCG is the primary lesion and aneurysmal bone cyst is a secondary change [20]. Thus whether the CGCG is solid or predominately cystic and hemorrhagic simply represents a different stage of the lesion evolution and the difference is likely related to the time of patient’s presentation. The USP6 gene rearrangement identified in our case is a further support to this speculation and the common pathogenesis as USP6 gene rearrangement has been documented to be present in most cases of primary aneurysmal bone cyst [21, 22]. USP6 gene rearrangement has been reported in CGCG previously [21] though our case is the first to report in gnathic location.

Management of CGCG is controversial and differs depending on the aggression, size, and location. Suggested treatment modalities in the literature include: surgery, radiation, interferon, intralesional steroids and tyrosine kinase inhibitors (Imatinib) [7, 23–26]. Of note, although calcitonin has also been suggested as a treatment modality, one randomized controlled trial found no improvement in results compared to placebo [27]. A combination of surgery and adjuvant interferon therapy has shown promise in another study specific for aggressive CGCG, with increased tumor control and decreased operative morbidity [28].

Surgery is the mainstay treatment of the aggressive variant of CGCG, once confirmed via adequate biopsy, typically performed in an operating room setting. Options include: enucleation, curettage and segmental resection. Enucleation and curettage are favourable due to the possible preservation of the cortex of the mandible and inferior alveolar nerve, however these methods are fraught with a recurrence rate of 30–70% in aggressive CGCG. According to one study, segmental resection has the lowest recurrence rate, specifically for lesions that present with pain, rapid growth, facial swelling, and cortical perforation [9, 29]. It is also pertinent to consider airway management for these cases, which may include a temporary tracheostomy as in our scenario.

For the patient presented here, segmental resection was felt to be the most likely treatment to avoid further recurrence and airway compromise. Although a controversial topic, due to the potential for impaired growth in the pediatric population and the possibility of poor function and cosmetic outcomes including malocclusion and asymmetry, this influenced the decision to proceed with titanium prosthesis implant versus vascularized free fibula flap reconstruction in our case [30].

## Conclusions

CGCG should be considered on the differential of any young child with a rapidly growing lesion of the mandible. Prompt diagnosis and management can greatly improve morbidity and long-term outcomes. Further studies are required to determine the optimal management for these lesions, particularly the aggressive variant of CGCG.

## Data Availability

Not applicable

## Abbreviations

CGCG: Central Giant Cell Granuloma
CHEO: Children’s Hospital of Eastern Ontario
CT: Computed Tomography
MRI: Magnetic Resonance Imaging
OMFS: Oral Maxillofacial Surgery
OR: Operating room
